# Chemical Structure of the Lipid A component of *Pseudomonas* sp. strain PAMC 28618 from Thawing Permafrost in Relation to Pathogenicity

**DOI:** 10.1038/s41598-017-02145-w

**Published:** 2017-05-19

**Authors:** Han-Gyu Park, Ganesan Sathiyanarayanan, Cheol-Hwan Hwang, Da-Hee Ann, Jung-Ho Kim, Geul Bang, Kyoung-Soon Jang, Hee Wook Ryu, Yoo Kyung Lee, Yung-Hun Yang, Yun-Gon Kim

**Affiliations:** 1 Department of Chemical Engineering, Soongsil University, Seoul, 06978 Korea; 2 Department of Biological Engineering, Konkuk University, Seoul, 05029 Korea; 3 Division of Bioconvergence Analysis, Korea Basic Science Institute, Chungbuk, 34133 Korea; 4 Division of Life Sciences, Korea Polar Research Institute, Incheon, 21990 Korea

## Abstract

Climate change causes permafrost thawing, and we are confronted with the unpredictable risk of newly discovered permafrost microbes that have disease-causing capabilities. Here, we first characterized the detailed chemical structure of the lipid A moiety from a *Pseudomonas* species that was isolated from thawing arctic permafrost using MALDI-based mass spectrometric approaches (i.e., MALDI-TOF MS and MALDI-QIT-TOF MS^n^). The MALDI multi-stage mass spectrometry (MS) analysis of lipid A extracted from the *Pseudomonas sp*. strain PAMC 28618 demonstrated that the hexaacyl lipid A ([M−H]^−^ at m/z 1616.5) contains a glucosamine (GlcN) disaccharide backbone, two phosphates, four main acyl chains and two branched acyl chains. Moreover, the lipid A molecule–based structural activity relationship with other terrestrial Gram-negative bacteria indicated that strain PAMC 28618 has an identical lipid A structure with the mesophilic *Pseudomonas cichorii* which can cause rot disease in endive (*Cichorium endivia*) and that their bacterial toxicities were equivalent. Therefore, the overall lipid A validation process provides a general strategy for characterizing bacteria that have been isolated from arctic permafrost and analyzing their respective pathogenicities.

## Introduction

The Arctic has experienced permafrost thawing due to a rapid temperature increase^1, 2^. Consequently, a number of native and undiscovered microorganisms in Arctic permafrost may be exposed and activated. The diversity of the microbial community across permafrost sites directly reflects the distinct conditions of the permafrost environment^2^. Such microorganisms could also utilize the ancient organic carbon in permafrost soils, which is thought to be mainly responsible for carbon dioxide and methane emissions^3^. Therefore, the characterizing the community structure and functional diversity of permafrost microbes is highly important for understanding the permafrost ecosystem and the emissions of greenhouse gasses in the permafrost. The isolation of permafrost bacteria and studies on permafrost microbes have increased recently^4–6^. The researchers who handle and study the unknown microbes, however, should be concerned about the pathogenicity of bacteria exposed from permafrost because some strains may cause unpredictable infectious diseases.

To screen and identify the endotoxins (i.e., lipopolysaccharide (LPS)) of unknown microbes, the most common approach is the Limulus Amebocyte Lysate (LAL) test^7^. In this assay, a proenzyme in LAL that is activated by the bacterial endotoxins catalyzes the cleavage of p-Nitroaniline (pNA) from a colorless peptide substrate. The quantity of released pNA increases proportionally with the endotoxin concentration. The LAL assay has generally been used to detect the endotoxin contamination in food, pharmaceutical and clinical specimens^8, 9^. Unfortunately, however, the results of the LAL test cannot directly link the presence of LPS to toxicity of lipid A from LPS^10, 11^. Therefore, more meticulous structural analysis of lipid A is required to determine the toxicity of newly discovered extremophile bacteria^12, 13^.

Lipopolysaccharides (LPS) are embedded in the external membranes of Gram-negative bacteria. These lipoglycans are virulence factors in the pathogenesis of host animal or plant cells^14, 15^. They have three major components: (1) the hydrophilic O-antigen, which is constitutes three to five repeating oligosaccharide subunits and serves as a major surface antigen; (2) the core oligosaccharide region, which contains a non-repeating short chain of sugar (e.g., 2-kedo-3-deoxy-octulonic acid) and maintains outer membrane stability; and (3) lipid A, which is the conserved amphipathic molecule anchored in the outer membrane. Importantly, the toxic activities of the Gram-negative bacteria that are associated with septic shock are mainly associated with the lipid A molecule^16^. However, there are multiple structural requirements for the toxicity of lipid A^17–19^. For example, lipid A that is toxic to animals or plants should contain all of the following structural components: (1) a glucosamine disaccharide, (2) diphosphorylation and (3) normal fatty acids^20^. Therefore, the lipid A from the newly discovered bacteria should be validated by a detailed structural analysis to understand the contribution of chemical changes to toxicity. To determine the detailed structure of lipid A, mass spectrometry (MS) has become a crucial technique because it allows the precise determination of the chemical structure and the molecular mass^21–30^. After the structural characterization of lipid A, the phenotypic toxicity of an invasive microorganism should be validated.

**Figure 1:**
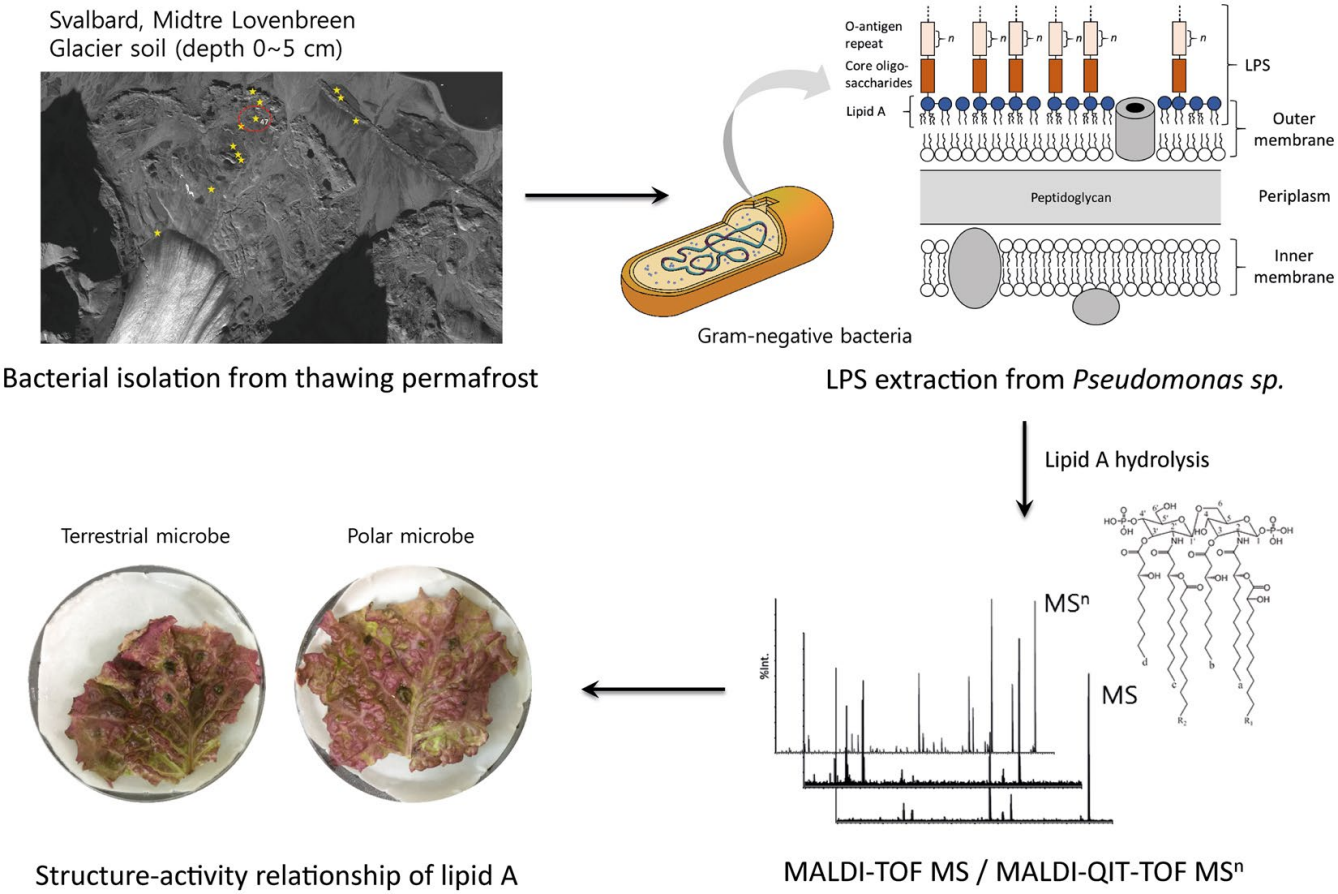
Overall workflow of the characterization of the lipid A molecule from polar Gram-negative bacteria by MALDI-based mass spectrometric approaches and the determination of pathogenical activity *via* structure-activity relationship.

In this study, we characterized the chemical structure of lipid A from an unknown artic bacterial isolate that was originally found in thawing permafrost and validated its phenotypical toxicity (Fig. 1). First, the newly screened arctic bacterium was identified using the 16S rRNA sequencing method as a *Pseudomonas sp*. (named *Pseudomonas sp*. strain PAMC 28618) and detected by the LAL assay. Then, MALDI-TOF MS and MALDI-QIT-TOF MS^n^ were applied to determine the partial and complete structures of the lipid A molecule. For this purpose, tandem MS which permits multiple stages of fragmentation (up to MS^5^) is essential for identifying more detailed structural information about the lipid A from Gram-negative bacteria. Through the multiple stages of fragmentation, we could precisely characterize the number of glucosamine (GlcN) and phosphate moieties, as well as the position of the ester-linked fatty acyl chains of lipid A. Interestingly, the chemical structure of lipid A from *Pseudomonas sp*. strain PAMC 28618 was identical to that of lipid A from *Pseudomonas cichorii* (ATCC 10857) which can cause rot disease in endive (*Cichorium endivia*). To verify the phenotypical toxicity of the *Pseudomonas* sp. strain PAMC 28618, the structure-activity relationship assay was performed with *P*. *cichorii*. This overall analytical process will be valuable for further polar microorganism research, and for studying the infectious diseases associated with newly discovered bacteria.

## Results and Discussion

### Taxonomic analysis and LAL assay

**Figure 2:**
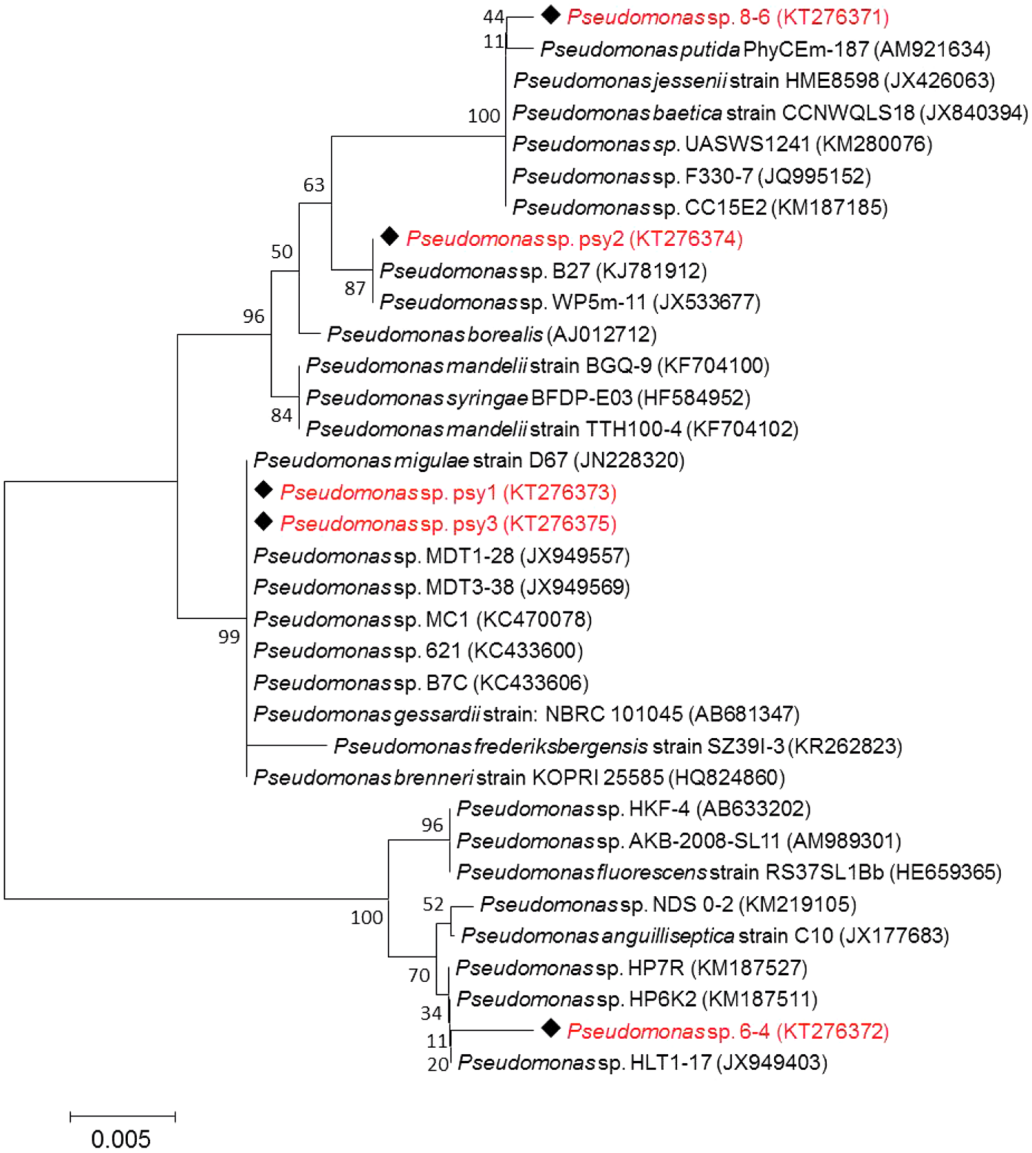
Neighbor-Joining (NJ) bootstrapping (100) phylogenetic tree of arctic glacier soil *Pseudomonas* spp. and their closest NCBI (BLASTn) strains based on the 16S rRNA gene sequences. Phylogenetic trees were developed based on the maximum composite likelihood method using MEGA 6.06 version.

Taxonomic analysis (BLASTn) of the five representative Arctic strains (PAMC 28615, 28616, 28617, 28618, and 28619) had shown a maximum similarity index for the Pseudomonas isolated from different environmental niches. Strains PAMC 28615, 28616, 28617, 28618, and 28619 had 99.9% sequence matches with *Pseudomonas* sp. 3–17 (KX378955), *Pseudomonas* sp. HP7R (KM187527), *Pseudomonas* sp. MDT3-38 (JX949569), *Pseudomonas frederiksbergensis* IHB B 10366 (KR233782), and *Pseudomonas* sp. JSZCNM3 (KU643204), respectively. The taxonomic relationship was confirmed with the *classifier* program of Ribosomal Database Project II release 11.4 (http://rdp.cme.msu.edu/). Therefore, 16S rRNA gene sequences and taxonomic relatedness have revealed that the isolated strains belong to genus *Pseudomonas*, and a similar group of organisms has been reported in Arctic and Antarctic glacier environments^31–33^. Typical homologous (98–99%) reference sequences were obtained from the *seqmatch* program of RDPII and used to construct the NJ phylogenetic tree (Fig. 2). Phylogenetic analysis showed that our Arctic pseudomonads sequences of 16S rRNA genes were matched to conserved regions of previously characterized 16S rRNA sequences of Arctic and Antarctic pseudomonads.

Previously, several studies have examined isolated bacteria in the Arctic and Antarctic soil to discover the microbial diversity^34–37^. However, the pathogenicity assay has not been actively performed with Arctic soil bacteria. As a model system, we focused on the toxicity of strain PAMC 28618 among the newly discovered bacteria in arctic soil, and an LAL assay was performed to investigate the presence of LPSs as endotoxins embedded in the external membrane of Gram-negative bacteria. The activity of endotoxins in the sonicated whole cell (strain PAMC 28618) was calculated using the standard calibration curve of endotoxin, which showed a correlation between the optical densities (OD) measured at 405 nm and endotoxin unit (EU) per mL (Fig. S3). As a result, the activity of LPS in *Pseudomonas* sp. strain PAMC 28618 (12 CFU) was calculated as approximatively 0.38 EU/mL. However, Takayama *et al*. reported that the positive LAL test does not directly correlate with its toxicity and there should be multiple structural requirements for the lipid A moiety: (1) a glucosamine disaccharide, (2) diphosphorylation, and (3) normal fatty acids^20^. To evaluate the toxicity of the newly discovered strain PAMC 28618, required a more detailed mass spectrometry-based structural analysis of lipid A, which is known to play a major role in the activity of endotoxins^38, 39^.

### MS analysis of lipid A from *Pseudomonas* sp. strain PAMC 28618 by MALDI-TOF MS

**Figure 3:**
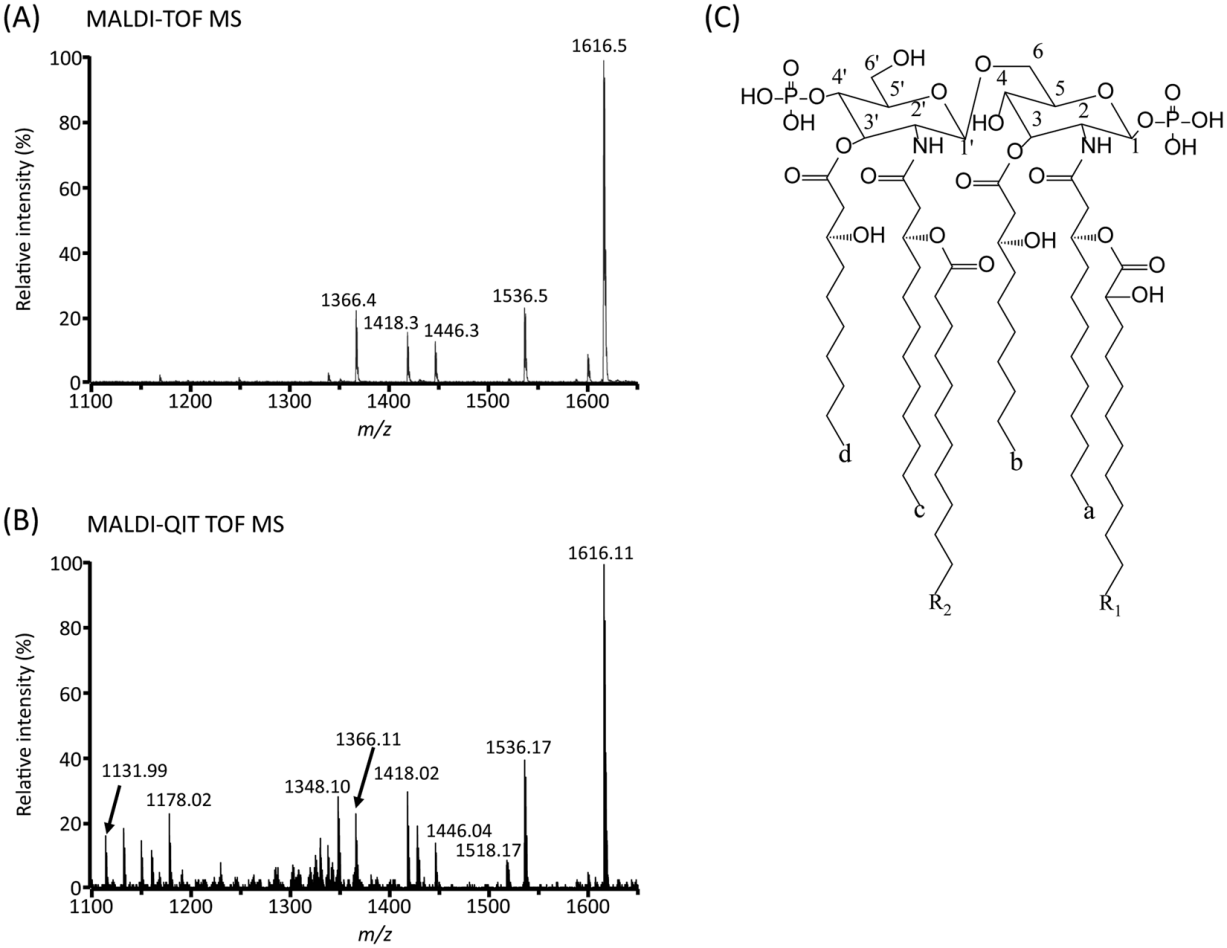
Negative-ion MALDI-TOF MS (**A**), MALDI-QIT TOF MS mass spectra of lipid A (**B**) and the lipid A molecule structure of *Pseudomonas* sp. strain PAMC 28618 (**C**).

The lipid A sample was prepared by hydrolysis of LPS that had been extracted from *Pseudomonas* sp. strain PAMC 28618 with phenol/water extraction^40^. First, to determine the parent ion mass of lipid A from strain PAMC 28618, the negative ion MALDI-TOF MS was applied. The MALDI-based analytical condition can generate more fragment ions of the lipid A molecule, which can provide structural information of the parent ion. The major peak at *m*/*z* 1616.5 is attributed to hexaacyl diphosphoryl lipid A which may contain two C10:0 (3-OH), two C12:0 (3-OH), one C12:0 (2-OH) and one C12:0 (Fig. 3C). The other peaks in the mass spectrum arise from fragmentation products of hexaacyl lipid A, where *m*/*z* 1536.5, 1446.3, 1418.3 and 1366.4 correspond to the elimination of the phosphate group at C-1, the acyl chain ‘d’ (C10:0 (3-OH)) at C-3′, the acyl chain ‘R_1_’ (C12:0 (2-OH)) at C-2 and both the acyl chain ‘d’ at C-3 and the phosphate group at C-1, respectively (Fig. 3A,B and Table S1). The intense peak at *m*/*z* 1536.5 is a product ion that is generated by the removal of the phosphate group at C-1 because the phosphate group at C-4′ may be more stable than that of C-1 due to the oxygen atom next to C-1^41^. With only the full mass spectrum of MALDI-TOF MS, we can sufficiently deduce the number of glucosamine (GlcN), phosphate groups and acyl chains of the lipid A molecule of strain PAMC 28618. However, to determine the structure of lipid A in more detail (i.e., the position and linkage of acyl chains and phosphates) from the tandem mass spectrometric analysis (MS^n^), MALDI-QIT TOF MS/MS was performed.

### MS analysis of the De-*O*-acylated lipid A from *Pseudomonas* sp. strain PAMC 28618 and *Pseudomonas cichorii* by MALDI-TOF MS

MALDI analysis of de-*O*-acylated lipid A can offer more detailed structural information about the fatty acid species and position (Fig. S4). To remove ester-linked fatty acids, mainly at C-3 and C-3′, lipid A was treated to mild alkaline condition as previously described^21, 42^. Silipo *et al*. reported *O*-acyl hydrolysis rate order that primary fatty acid is hydrolyzed first^21^. Thus, a peak at m/z 1276.0 is readily explained by the absence of the ester-linked primary fatty acids (C10:0 (3-OH) ‘b’ and ‘d’) at the C-3 and C-3′ position. Additionally, a peak at m/z 1247.8 and 1013.8 indicates the presence of secondary fatty acid R_1_ and R_2_ linked N-acyl residue, respectively (Fig. S4). However, the position of the secondary fatty acid was not identified. Thus, tandem mass spectrometric analysis *via* MALDI-QIT TOF MS was performed to determine the location of the secondary fatty acid.

### MS^n^ analysis of lipid A from *Pseudomonas* sp. strain PAMC 28618 by MALDI-QIT TOF MS

**Figure 4:**
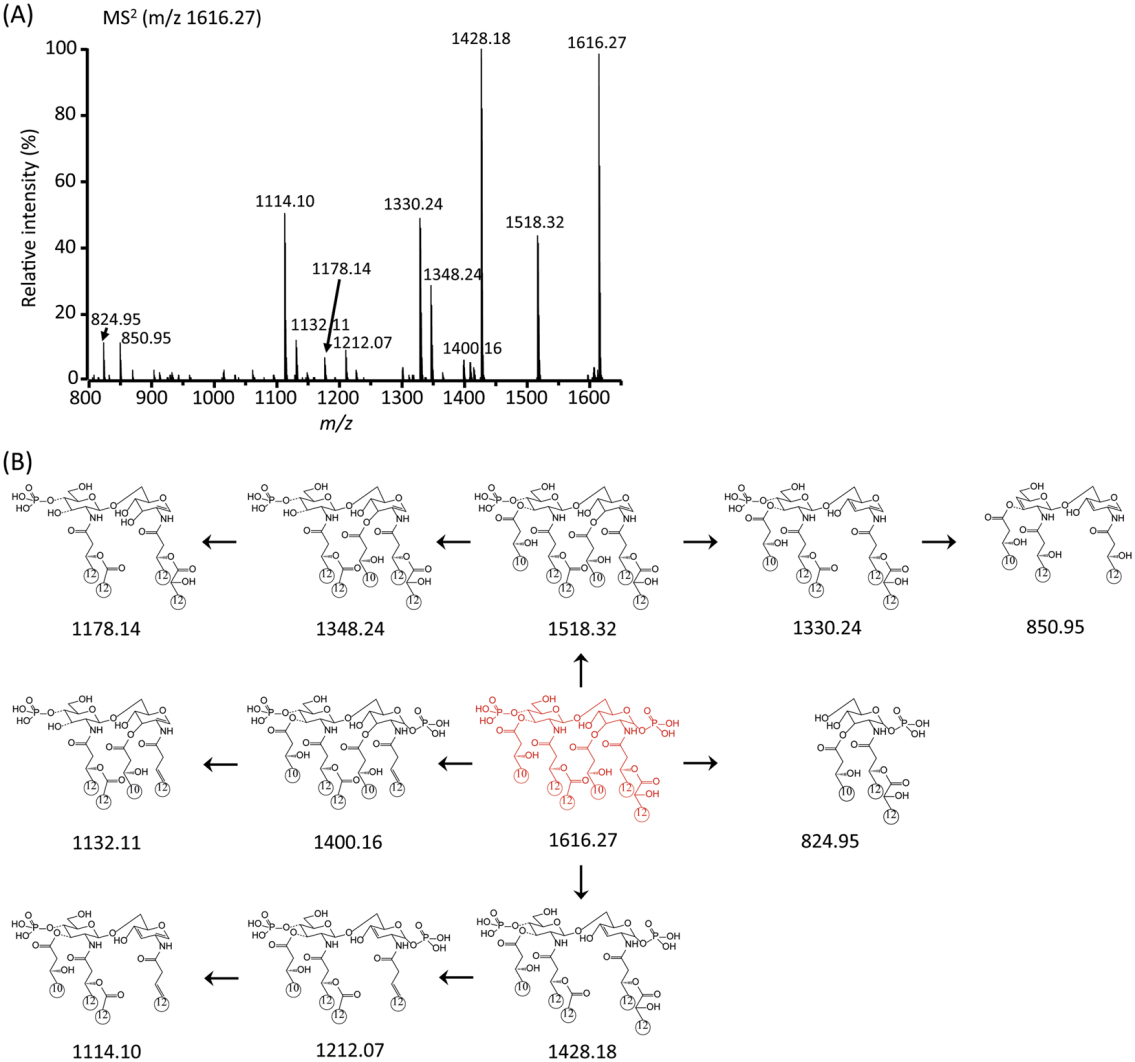
Negative-ion MALDI-QIT-TOF MS^2^ spectrum of parent peak at *m/z* 1616.27 (**A**) and fragment structures corresponding to each fragment ion peak were drawn as neutral molecules (**B**).

First, the MALDI-QIT TOF mass spectrum exhibited the prominent fragmental ions of lipid A of *Pseudomonas* sp. strain PAMC 28618, which were detected by MALDI-TOF MS analysis. With the full mass MALDI-TOF and MALDI-QIT TOF mass spectra, we can confirm that the parent ion of lipid A from strain PAMC 28618 is at 1616.11 [M−H]^−^. To characterize the detailed chemical structure of lipid A, we conducted a multiple stages of fragmentation (up to MS^5^) study and selected the parental ion at m/z 1616.27 in the first MS/MS stage. In the MS^2^ spectrum, according to Luisa Sturiale *et al*., a peak at m/z 824.95 was identified as a Y-type fragment ion of GlcN I *via* in-source decay^25^. Thus, the peak can be unequivocally explained by the presence of the secondary fatty acid C12:0 (2-OH) on N-linked to GlcN I. Additionally, the ion at m/z 850.95 indicated the presence of the secondary fatty acid C12:0 linked amide primary fatty acid C12:0 of GlcN II. The major fragment peaks were observed at m/z 1518.32, 1428.18, 1330.24 and 1114.10 corresponding to the cleavage of the phosphate group at C-1, ‘b’ acyl chain at C-3, the phosphate group and ‘b’ acyl chain at C-3, and the phosphate and two acyl groups ‘b’ at C-3 and ‘R_1_’ at C-2, respectively (Fig. 4 and Table S2). Interestingly, in the intense peak at m/z 1428.18, we deduced that the fragment peak was generated by the elimination of the acyl chain ‘b’, but not ‘d’. This is because the ion at m/z 808.96 can only be detected when the ‘d’ acyl chain remained at C-3′ during the MS^3^ stage of m/z 1428.19 ions (Fig. S5 and Table S6). The QIT MS^2^ fragmentation patterns were quite different from the MALDI-MS fragments, which means that each MS^n^ stage will provide more detailed structural information for characterizing the lipid A of strain PAMC 28618. The highly abundant ions at m/z 1518.32 and 1428.18 were isolated for the MS^3^ stage for further structural characterization.

**Figure 5:**
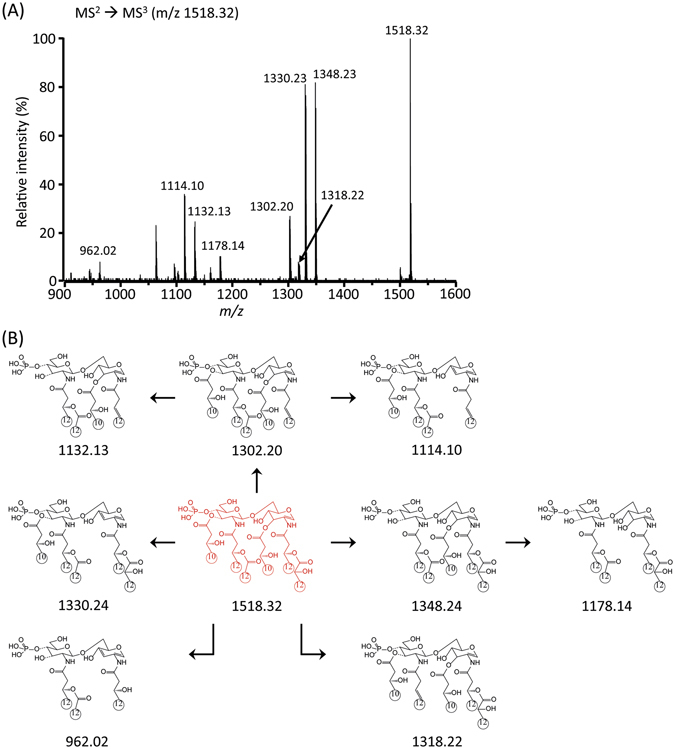
Negative-ion MALDI-QIT-TOF MS^3^ spectrum of monophosphoryl lipid A at *m/z* 1518.32 (**A**) and fragment structures (**B**).

In the MS^3^ stage, the product ions of m/z 1348.24, 1330.24 and 1302.20 were generated by eliminating a ‘d’ acyl chain at C-3′, ‘b’ acyl chain at C-3 and ‘R_1_’ at C-2 from m/z 1518.32 as shown in Fig. 5A and Table S3. Two fragmentation mechanisms with CID (collision-induced dissociation) might be applied to the product ions at m/z 1348.24 and 1330.24. First, the charge-driven process resulted in the neutral loss of an acyl chain as a ketone derivative from the parent ion at C-3′. Next, the charge-remote process led to the neutral loss of an acyl chain as a free fatty acid at C-3. When we compare the product ion peak intensities, the results suggest that the charge-driven and charge-remote fragmentation processes compete with each other in the CID experiments using MALDI-QIT-TOF MS (Figs 5B and S2).

**Figure 6:**
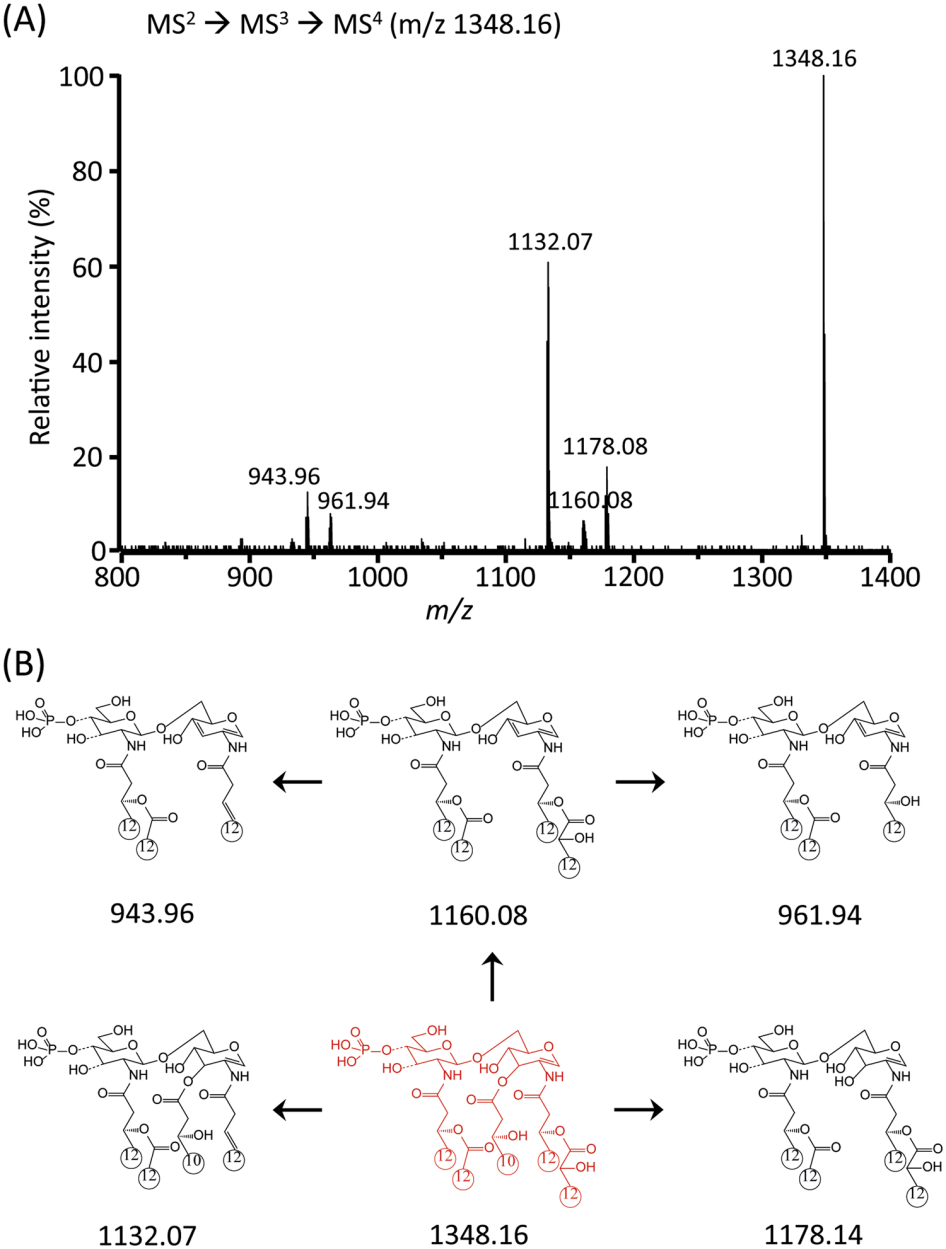
Negative-ion MALDI-QIT-TOF MS^4^ spectrum of *m/z* 1348.16 corresponding to removal of acyl group at C-3′ (**A**) and fragment structures (**B**).

**Figure 7:**
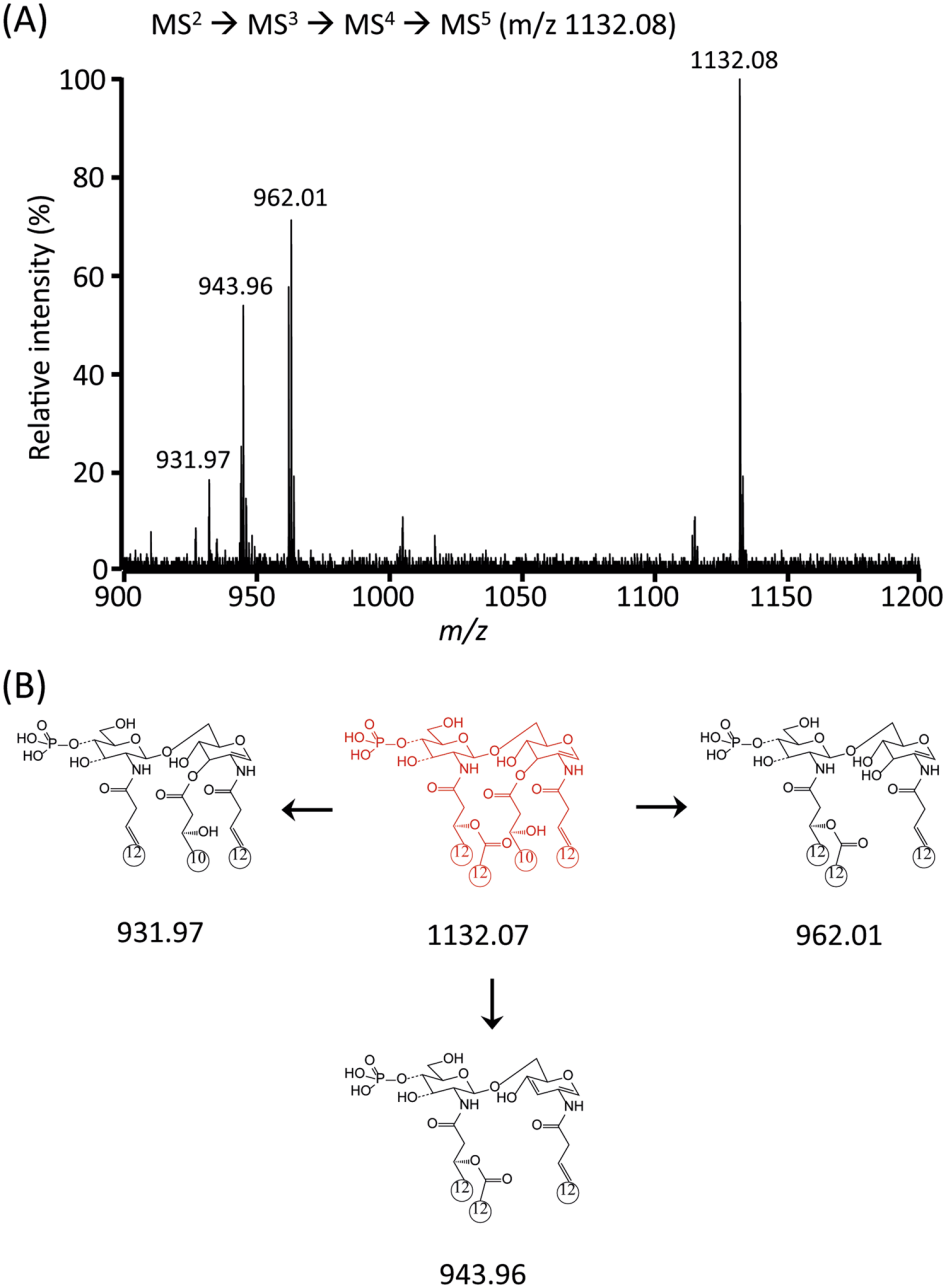
Negative-ion MALDI-MS^5^ spectrum of *m/z* 1132.08 (**A**) and fragment structure (**B**).

The abundant product ions at m/z 1348.23 (Fig. 5A) and 1330.24 (Fig. S5A) from the MS^3^ stage were isolated for the MS^4^ stage (Figs 6 and S6; Tables S4 and S). The highest fragment ion at m/z 1132.07 corresponded to the elimination of ‘R_1_’ at C-2 from m/z 1348.16. The other fragment ions clearly supported the chemical structure of the parent ion at m/z 1348.24. Finally, the ion at m/z 1132.07 from the MS^4^ stage was selected for the subsequent MS^5^ analysis. The existence of the free fatty acid (C10:0 (3-OH)) at C-3 was confirmed by the formation of an ion at m/z 943.96 and 962.01 by MS^5^ (Figs 7 and S7; Tables S5 and S8). Taken together, the tandem mass analysis of lipid A from strain PAMC 28618 demonstrated the exact chemical structure of hexaacyl diphosphoryl lipid A. The two phosphate groups are located at the C-1 and C-4′ positions of the glucosamine dimer. In addition, two C10:0 (3-OH) acyl chains are at the C-3 and C-3′ positions *via* ester bonds and two C12:0 (3-OH) acyl chains are at C-2 and C-2′ *via* amide bonds. The two branched chains (i.e., C12:0 and C12:0 (2-OH)) are substituted at the main chains of C-2 and C-2′, respectively.

### Structure-activity relationship of lipid A from *Pseudomonas* sp. strain PAMC 28618 with *Pseudomonas cichorii*

After we had characterized the chemical structure of lipid A from *Pseudomonas* sp. strain PAMC 28618, we were curious whether any mesophilic *Pseudomonas* sp. has the same chemical structure of lipid A. Interestingly, Molinaro *et al*. reported the structure of the lipid A fraction from LPS of the *Pseudomonas cichorii* using 2D-NMR and MALDI-TOF MS^43^. According to our taxonomic analysis (BLASTn), *P. cichorii* had 97% sequence matches with strain PAMC 28618. The intact lipid A from *P. cichorii* also consisted of glucosamine disaccharides, two C12:0 (3-OH) at C-2 and C-2′, and two C10:0 (3-OH) at C-3 and C-3′. C12:0 (2-OH) and C12:0 are linked to both C12:0 (3-OH) at C-2 and C-2′. Finally, each phosphate group is linked to C-1 and C-4′. Taken together, the overall structure of lipid A from *P*. *cichorii* is identical to that from *Pseudomonas sp*. strain PAMC 28618. To validate the chemical structure of lipid A from *P. cichorii* which was determined by 2D-NMR and MALDI-TOF MS^43^, we conducted multiple-stage MS analysis of lipid A from *P. cichorii* (Figs S8–11, Tables S9–12). With the full MALDI-TOF and MALDI-QIT TOF mass spectra, we can confirm that the parent ion of lipid A of *P*. *cichorii* is at 1616.10 [M−H]^−^. Moreover, the MS^n^ analysis of lipid A from *P*. *cichorii* demonstrated that the lipid A of both *P. cichorii* and strain PAMC 28618 has an identical fine structure. The Gram-negative bacterium *Pseudomonas cichorii* has been identified to cause a destructive disease known as “varnish spot” on lettuce, cabbage, celery and chrysanthemum plants^44, 45^. Previous studies have revealed that LPS plays an important role in the pathogenesis of Gram-negative bacterial infection, and that its endotoxic effects such as cytokine production, inflammation and shock are largely determined by its lipid A^46^. Therefore, based on the structural identity between *P. cichorii* and strain PAMC 28618, we investigated the bacterial toxicity of the newly discovered polar microorganism.

**Figure 8:**
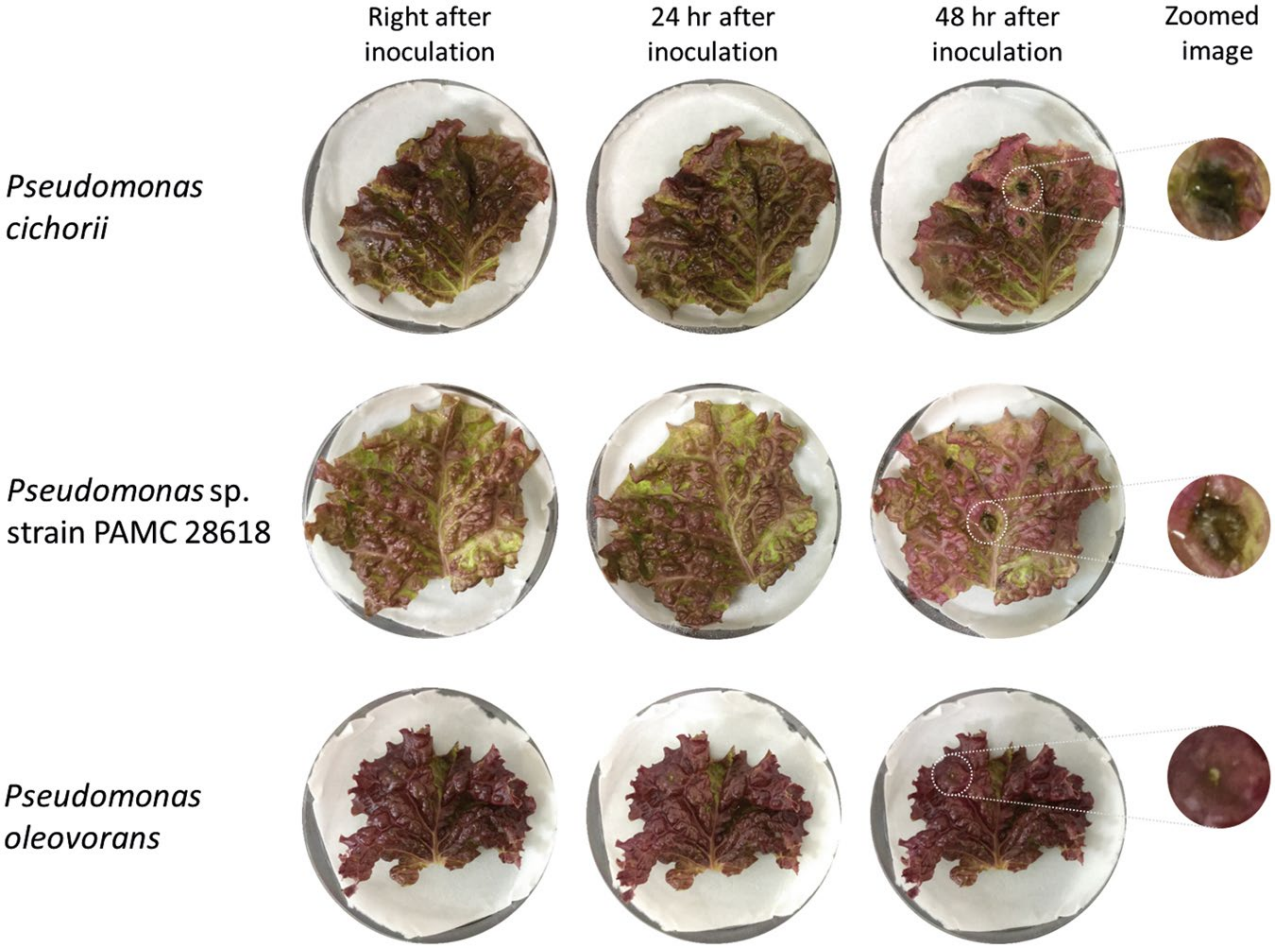
Phenotypical toxicity test was performed by inoculating lettuce leaves with *Pseudo- monas cichorii*, strain PAMC 28618 or *P. oleovorans* as a control and leaves were observed every 24 hr for 48 hr. After 48 h, the spot that had been inoculated with *P. cichorii* and strain PAMC 28618 were showed rot disease, but lettuce inoculated with *P. oleovorans* did not develop rot disease.

Lettuce that had been cultured at regulated conditions (temperature = 20 °C, humidity = 50% and pH = 6.0 to 7.0) was inoculated with a high population density of *P. cichorii*, *Pseudomonas* sp. strain PAMC 28618 and *P. oleovorans* (94 × 10^4^, 54 × 10^4^ and 44 × 10^5^ CFU) to verify proof-of-concept with real plant sample*. Pseudomonas oleovorans* was used as a control (Fig. 8). Inoculation was conducted using pipette tips to prick a hole in the leaves on Whatman chromatography paper in a petri dish. This inoculation process was slightly modified from the work of Dong *et al*.^47^.

After 24 hours, we could identify symptoms in the early stage of the disease at inoculated spots with strain PAMC 28618 and *P. cichorii*. After 48 hours, necrotic dark-brown areas were clearly observed and were larger than the brown areas recorded in the same spots at 24 hours. As expected, there were no symptoms at the spot inoculated with *Pseudomonas oleovorans*. Therefore, the newly screened polar microorganism has an identical lipid A structure as the mesophilic *P. cichorii* and their bacterial toxicity was equivalent. To the best of our knowledge, this is the first report to identify the relationship between the lipid A molecule structure of a polar microbe and its toxicity. However, further studies may be needed to demonstrate that all Gram-negative bacteria with the same lipid A structure show similar toxicity.

## Conclusions

In this study, we characterized the chemical structure of the lipid A moiety from *Pseudomonas* sp. strain PAMC 28618 which was newly discovered in thawing arctic soils using mass spectrometric approaches with MALDI-TOF and MALDI-QIT-TOF MS. Based on the structural relationship of lipid A, we directly compared the phenotypical toxicity of strain PAMC 28618 with *P. cichorii*. During the multi-stage tandem mass spectrometry (MS^5^), the diagnostic fragment ions were used to characterize the hexaacyl diphosphoryl lipid A from strain PAMC 28618. Although the genotypical (16S rRNA sequencing) characterization of strain PAMC 28618 was not completely identical to that of other terrestrial Gram-negative bacteria, the structural relationships of lipid A may be significantly correlated with the bacteria’s phenotypic toxicities. It is believed that the overall lipid A analytical process would be valuable for studying the newly discovered Arctic bacteria which are not free of diseases, and for tackling critical and largely unmet challenges in polar microbiology.

## Materials and Methods

### Site description and sample collection

The sampling site is located in the glacier foreland of Midtre Lovenbreen, Svalbard (78.9°N, 12.0°E). The field campaign was permitted by the Svalbard Science Forum (RIS ID: 6752) in July, 2014. The glacier foreland soil samples were collected from different sites (Fig. S1) at 0~5 cm depth, placed in sterile 50 mL Falcon tubes and the samples were stored at 5 °C in the Arctic. Then, the samples were transported to the laboratory in ice buckets, containing ice packs that had been precooled to −70 °C and were stored at 4 °C until they were processed for bacterial isolation.

### Isolation and culturing of Arctic pseudomonads

Approximately 1 g of glacier foreland soil sample was inoculated into 10 mL of Nutrient Broth (Acumedia, Lansing, MI, USA) and 10 mL of lysogeny broth (LB) (Merck KGaA, Darmstadt, Germany)^48^. The inoculated media were incubated at 20 °C for 24 hr under aerobic condition. The enriched culture was then serially diluted and 100 μl was plated on Pseudomonas Isolation Agar (17208, Sigma-Aldrich, St. Louis, MO, USA) plates (Peptic digest of animal tissue, 20 g/L; Potassium sulfate, 10 g/L; Magnesium chloride, 1.4 g/L; Triclosan (Irgasan), 0.025 g/L; Agar, 13.6 g/L; pH, 7.0 ± 0.2). Agar plates were incubated at 4, 15, 20 and 25 °C for two weeks under aerobic condition. Single pseudomonads colonies were obtained and each colony was repeatedly plated on Pseudomonas Agar (P2102, Sigma-Aldrich, St. Louis, MO, USA) to obtain pure isolated aerobic pseudomonads. Pure cultures were stored at 4 °C and cryopreserved in 20% (v/v) glycerol at −70 °C. Subsequently, Arctic pseudomonad strains (8–6, 6–4, psy1, psy2, and psy3) were deposited into the Korea Polar Research Institute (KPRI) based Polar and Alpine Microbial Collection (PAMC) center under the general deposit category with accession numbers PAMC 28615 to PAMC 28619, respectively.

### DNA extraction, 16S rRNA gene amplification, and phylogenetic analysis

Genomic DNA was extracted from the five Arctic pseudomonads, and 16S rRNA gene was amplified by polymerase chain reaction (PCR) using universal degenerate primers 27 F (5′-AGA GTT TGA TCC TGG CTC AG-3′) and 1492 R (5′-GGT TAC CTT GTT ACG ACT T-3′) as previously described^49^. The PCR amplicon was cloned using TOPO TA cloning kit (Invitrogen, CA, USA) and then sequenced. 16S rRNA gene sequences obtained from the strains PAMC 28615 to PAMC 28619 were compared with those of other pseudomonads using NCBI BLAST (http://blast.ncbi.nlm.nih.gov/Blast.cgi) for their pair-wise identities. Multiple sequence alignment was carried out using the ClustalW2 version of EBI (http://www.ebi.ac.uk/Tools/msa/clustalw2/) with 0.5 transition weight. Phylogenetic trees were constructed using the MEGA software version 6.06 (http://www.megasoftware.net) by neighbor joining (NJ) with the Kimura two-parameter model. Moreover, the Ribosomal database project (RDP-11) (https://rdp.cme.msu.edu/) was also used to determine the strain identity with a seqmatch and classifier program. The 16S rRNA gene sequences of the Arctic pseudomonads were deposited in the GenBank nucleotide sequence database under the following accession numbers: PAMC 28615 (KT276371), PAMC 28616 (KT276372), PAMC 28617 (KT276373), PAMC 28618 (KT276374), and PAMC 28619 (KT276375).

### Extraction of LPS from *Pseudomonas* sp. strain PAMC 28618 and *Pseudomonas cichorii*

*Pseudomonas* sp. strain PAMC 28618 and *Pseudomonas cichorii* was grown at 30 °C in lysogeny broth (LB) and Nutrient broth (NB), respectively, with incubation for 24 hr at 250 rpm in a shaking incubator. A 2% (v/v) of bacterial culture was used as the inoculum for culturing in a 100 mL Erlenmeyer flask.

LPS extraction from the cultured cells was performed using an LPS extraction kit (Intron Biotechnology Inc., Sungnam, Korea). First, 10 mL of lysis buffer containing phenol from the kit was added to 25 mg of the cell and vigorously vortexed to improve the lysis of the bacterial cell. After 2 mL of chloroform was added to the lysis buffer, it was vortexed for 30 sec. The mixture was then centrifuged at 13000 rpm and 4 °C for 10 min. Subsequently, 4 mL of supernatant was transferred to a new 50 mL Falcon tube and 8 mL of purification buffer was added. The mixture was incubated for 30 min at −20 °C to purify the LPS of other cell extract components, such as protein, nucleic acid, and lipids, and it was then centrifuged at 13,000 rpm and 4 °C for 20 min. After 8 mL of supernatant had been transferred to a new 50 mL Falcon tube, the LPS pellet was obtained by centrifugation under previously described conditions. The extracted LPS was washed with 10 mL of 70% EtOH and completely dried. Through this extraction process, 2.1 mg and 2.2 mg of LPS were extracted from 25 mg of strain PAMC 28618 and *P. cichorii*, respectively.

### Lipid A extraction

The LPS pellet was suspended in 200 μl of 1% acetic acid. It was denatured for 2 hr at 100 °C and lipid A was extracted using the chloroform methanol extraction method. The chloroform methanol extraction process was performed by mixing 200 μl of the solution with 400 μl of chloroform and 200 μl of distilled water. The mixed solution was centrifuged at 8000 rpm for 15 min at 15 °C. The bottom layer was transferred to a new tube and dried by nitrogen gas.

### De-*O*-acylation of lipid A

Extracted lipid A was de-*O*-acylated with 200 μl of 1:3 diluted ammonium hydroxide hydrolysis for 16 h at room temperature and it was dried under nitrogen gas as previously reported^21^.

### MALDI-TOF MS and MALDI-QIT-TOF MS^n^ analysis

Dried lipid A samples were dissolved with 10 μl of 50% (v/v) methanol/chloroform. Then, 1 μl of the lipid A sample was mixed with 1 μl of 50 mg 2,5-dihydroxybenzoic acid (DHB) solution in 1 mL 70% (v/v) acetonitrile/water. Subsequently, 2 μl of the mixture was loaded on a MSP 96 target polished steel MALDI plate and dried at room temperature. The lipid A samples were analyzed via a Bruker Daltonics Microflex LRF MALDI-TOF MS instrument and controlled by FlexControl 3.0 software (Bruker, Bremen, Germany). The operating conditions were as follows: negative-ion and reflectron mode, detector gain = 6.9, laser frequency = 60.0 Hz and laser power = 70%. Data acquisition and processing were performed by Flexanalysis 3.3 software (Bruker, Bremen, Germany). MS/MS analysis was performed using an Axima Resonance MALDI-quadrupole ion trap-TOF instrument (Shimadzu, Manchester, UK). The instrument was operated in negative ion and reflectron mode. Fragment ions were analyzed by collision-induced dissociation (CID) of the precursor ions and argon gas was used as a collision gas. Operating conditions were as follows: intro endcap = −5.5 kV, extr endcap = 10.0 kV, flight tube = 10.0 kV, reflectron center = −0.2 kV, reflectron back = −0.2 kV, and Detector = 2.2 kV. The data acquisition and processing were performed using the Launchpad 2.9.3 software (Kratos Analytical, Manchester, UK).

### LAL (Limulus amebocyte lysate) assay

The LAL assay was performed using LAL Chromogenic Endotoxin Quantitation Kit (Thermo Scientific Pierce, Rockford, IL, USA) to confirm the existence of LPS in Pseudomonas strain PAMC 28618. The assay kit was verified to be working using standard endotoxin (0.1 to 1.0 EU/mL). Then, the LAL assay was performed using serially diluted cell lysate (12 CFU) of strain PAMC 28618 and according to the manufacturer’s protocol.

### Inoculation of bacteria in lettuce for pathological test

*Pseudomonas* sp. strain PAMC 28618 and *Pseudomonas oleovorans* were cultured in lysogeny broth (LB) and the *P. cichorii* strain was cultured in Nutrient broth (NB). All three strains were cultured at 30 °C for 24 hr. The lettuce was grown for 2~3 weeks under controlled conditions. Regulated conditions were as follows: temperature = 20 °C, humidity = 50% and pH = 6.0 to 7.0. The lettuce was placed in Petri dishes containing Whatman Chromatography paper drenched with sterile water (3 mL) to create a damp environment. A volume of 20 μl of the cultured bacteria was used to inoculate the punched lettuce leaves with the end of the pipette tip beforehand. Then, the Petri dishes were placed in a 30 °C incubator and evaluated every 24 hr for 48 hr.

## Electronic supplementary material

Supplementary Information
