# Supplementary material for: Chemical Structure of the Lipid A component of Pseudomonas sp. strain PAMC 28618 from Thawing Permafrost in Relation to Pathogenicity
Source: Sci Rep. 2017 May 19;7:2168. doi: 10.1038/s41598-017-02145-w (PMC5438365; doi:10.1038/s41598-017-02145-w)

Supplementary Information (SI)

Chemical Structure of the Lipid A component of *Pseudomonas* sp. strain PAMC 28618 from Thawing Permafrost in Relation to Pathogenicity

Han-Gyu Park[1], Ganesan Sathiyanarayanan[2], Cheol-Hwan Hwang[1], Da-Hee Ann[1], Jung-Ho Kim[2], Geul Bang[3], Kyoung-Soon Jang[3], Hee Wook Ryu[1], Yoo Kyung Lee[4,*], Yung-Hun Yang[2,*], Yun-Gon Kim[1,*]

[1]*Department of Chemical Engineering, Soongsil University, Seoul 06978, Korea*

[2]*Department of Biological Engineering, Konkuk University, Seoul 140-701, Korea*

[3]*Division of Bioconvergence Analysis, Korea Basic Science Institute, Chungbuk 34133, Korea*

[4]*Korea Polar Research Institute, Incheon 21990, Korea*

Supplementary Tables

Table S1. The molecular masses of the lipid A from *Pseudomonas sp.* strain PAMC 28618 and its fragmentation from negative ion MALDI-TOF MS

	Observed mass[a] (m/z)	Calculated mass[a] (m/z)	Interpretation of peaks[b]
MALDI-TOF MS	1616.5	1616.0	$[M - H]^-$
	1536.5	1536.0	$[1616.5 - PO_3H]^-$
	1446.3	1445.9	$[1616.5 - C_{10}H_{18}O_2]^-$
	1418.3	1417.8	$[1616.5 - C_{12}H_{22}O_2]^-$
	1366.4	1365.9	$[1536.5 - C_{10}H_{18}O_2]^-$

[a] Observed mass and calculated mass are the form of $[M\text{-}H]^-$

[b] M, intact lipid A.

Table S2. Observed peaks in the negative MALDI-QIT TOF spectrum on MS^2 of m/z 1616.27

	Observed mass (m/z)	Calculated mass (m/z)	Interpretation of peaks
MS/MS of m/z 1616.27 parent ion	1616.27	1615.99	$[M - H]^-$
	1518.32	1518.01	$[1616.27 - PO_4H_3]^-$
	1428.18	1427.85	$[1616.27 - C_{10}H_{20}O_3]^-$
	1400.16	1399.82	$[1616.27 - C_{12}H_{24}O_3]^-$
	1348.24	1347.88	$[1518.32 - C_{10}H_{18}O_2]^-$
	1330.24	1329.87	$[1518.32 - C_{10}H_{20}O_3]^-$
	1212.07	1211.67	$[1428.18 - C_{12}H_{24}O_3]^-$
	1178.14	1177.75	$[1348.24 - C_{10}H_{18}O_2]^-$
	1132.11	1131.71	$[1400.16 - C_{10}H_{18}O_2 - PO_4H_3]^-$
	1114.10	1113.70	$[1212.07 - PO_4H_3]^-$
	850.95	851.56	$[1330.24 - C_{12}H_{22}O - C_{12}H_{22}O_2 - PO_4H_3]^-$
	824.95	824.49	$[M - C_{40}H_{74}NO_{12}P - H]^-$

Table S3. Observed peaks in the negative MALDI-QIT TOF spectrum on MS^3 of m/z 1518.32

	Observed mass (m/z)	Calculated mass (m/z)	Interpretation of peaks
MS^3 of m/z 1518.32 parent ion	1348.23	1347.88	$[1518.32 - C_{10}H_{18}O_2]^-$
	1330.23	1329.87	$[1518.32 - C_{10}H_{20}O_3]^-$
	1318.22	1317.83	$[1518.32 - C_{12}H_{24}O_2]^-$
	1302.20	1301.84	$[1518.32 - C_{12}H_{24}O_3]^-$
	1178.14	1177.75	$[1348.23 - C_{10}H_{18}O_2]^-$
	1132.13	1131.71	$[1302.20 - C_{10}H_{18}O_2]^-$
	1114.10	1113.70	$[1302.20 - C_{10}H_{20}O_3]^-$
	962.02	961.58	$[1330.23 - C_{10}H_{18}O_2 - C_{12}H_{22}O_2]^-$

Table S4. Observed peaks in the negative MALDI-QIT TOF spectrum on MS^4 of m/z 1348.16

	Observed peaks (m/z)	Calculated peaks (m/z)	Interpretation of peaks
MS^4 of m/z 1348.16 parent ion	1178.08	1177.75	$[1348.16 - C_{10}H_{18}O_2]^-$
	1160.08	1159.74	$[1348.16 - C_{10}H_{20}O_3]^-$
	1132.07	1131.71	$[1348.16 - C_{12}H_{24}O_3]^-$
	961.94	961.58	$[1160.08 - C_{12}H_{22}O_2]^-$
	943.96	943.57	$[1160.08 - C_{12}H_{24}O_3]^-$

Table S5. Observed peaks in the negative MALDI-QIT TOF spectrum on MS^5 of m/z 1132.08

	Observed peaks (m/z)	Calculated peaks (m/z)	Interpretation of peaks
MS^5 of m/z 1132.08 parent ion	962.01	961.58	$[1132.08 - C_{10}H_{18}O_2]^-$
	943.96	943.57	$[1132.08 - C_{10}H_{20}O_3]^-$
	931.97	931.53	$[1132.08 - C_{12}H_{24}O_2]^-$

Table S6. Observed peaks in the negative MALDI-QIT-TOF spectrum on MS^3 of m/z 1428.19

	Observed peaks (m/z)	Calculated peaks (m/z)	Interpretation of peaks
MS^3 of m/z 1428.19 parent ion	1330.24	1329.87	$[1428.19 - PO_4H_3]^-$
	1212.07	1211.67	$[1428.19 - C_{12}H_{24}O_3]^-$
	1178.15	1177.75	$[1428.19 - C_{10}H_{18}O_2\text{-}PO_3H]^-$
	1132.08	1131.71	$[1330.24 - C_{12}H_{22}O_2]^-$
	1114.12	1113.70	$[1212.07 - PO_4H_3]^-$
	1016.11	1015.72	$[1114.12 - PO_4H_3]^-$
	930.94	931.53	$[1132.08 - C_{12}H_{24}O_2]^-$
	850.96	851.56	$[930.94 - PO_3H]^-$
	808.96	808.50	$[1428.19 - C_{30}H_{54}NO_{10}P]^-$

Table S7. Observed peaks in the negative MALDI-QIT-TOF spectrum on MS^4 of m/z 1330.24

	Observed peaks (m/z)	Calculated peaks (m/z)	Interpretation of peaks
MS^4 of m/z 1330.24 parent ion	1160.17	1159.74	$[1330.24 - C_{10}H_{18}O_2]^-$
	1130.12	1129.69	$[1330.24 - C_{12}H_{24}O_2]^-$
	1114.11	1113.70	$[1330.24 - C_{12}H_{24}O_3]^-$
	1052.12	1051.74	$[1330.24 - C_{12}H_{22}O_2\text{-}PO_3H]^-$
	962.01	961.58	$[1160.17 - C_{12}H_{22}O_2]^-$

Table S8. Observed peaks in the negative MALDI-QIT-TOF spectrum on MS^5 of m/z 1114.13

	Observed peaks (m/z)	Calculated peaks (m/z)	Interpretation of peaks
MS^5 of m/z 1114.13 parent ion	1016.19	1015.72	$[1114.13 - PO_4H_3]^-$
	944.03	943.57	$[1114.13 - C_{10}H_{18}O_2]^-$
	926.02	925.56	$[1114.13 - C_{10}H_{20}O_3]^-$
	913.97	913.52	$[1114.13 - C_{12}H_{24}O_2]^-$

Table S9. The molecular masses of the lipid A from *Pseudomonas cichorii* and its fragmentation from negative ion MALDI-TOF MS

	Observed peaks (m/z)	Calculated peaks (m/z)	Interpretation of peaks
MALDI-TOF MS	1616.2	1616.0	$[M - H]^-$
	1536.2	1536.0	$[1616.2 - PO_3H]^-$
	1446.0	1445.9	$[1616.2 - C_{10}H_{18}O_2]^-$
	1418.0	1417.8	$[1616.2 - C_{12}H_{22}O_2]^-$
	1366.1	1365.9	$[1536.2 - C_{10}H_{18}O_2]^-$

Table S10. Observed peaks in the negative MALDI-QIT-TOF spectrum on MS^2 of m/z 1616.10

	Observed peaks (m/z)	Calculated peaks (m/z)	Interpretation of peaks
MS/MS of m/z 1616.10 parent ion	1616.10	1615.99	$[M - H]^-$
	1518.19	1518.01	$[1616.10 - PO_4H_3]^-$
	1428.07	1427.85	$[1616.10 - C_{10}H_{20}O_3]^-$
	1400.01	1399.82	$[1616.10 - C_{12}H_{24}O_3]^-$
	1348.15	1347.88	$[1518.19 - C_{10}H_{18}O_2]^-$
	1330.15	1329.87	$[1518.19 - C_{10}H_{20}O_3]^-$
	1212.00	1211.67	$[1428.07 - C_{12}H_{24}O_3]^-$
	1178.00	1177.75	$[1348.15 - C_{10}H_{18}O_2]^-$
	1132.06	1131.71	$[1400.01 - C_{10}H_{18}O_2 - PO_4H_3]^-$
	1114.06	1113.70	$[1212.00 - PO_4H_3]^-$
	850.94	851.56	$[1330.15 - C_{12}H_{22}O - C_{12}H_{22}O_2 - PO_4H_3]^-$
	824.94	824.49	$[M - C_{40}H_{74}NO_{12}P - H]^-$

Table S11. Observed peaks in the negative MALDI-QIT-TOF spectrum on MS^3 of m/z 1518.19

	Observed peaks (m/z)	Calculated peaks (m/z)	Interpretation of peaks
MS^3 of m/z 1518.19 parent ion	1348.15	1347.88	$[1518.19 - C_{10}H_{18}O_2]^-$
	1330.14	1329.87	$[1518.19 - C_{10}H_{20}O_3]^-$
	1302.12	1301.84	$[1518.19 - C_{12}H_{24}O_2]^-$
	1178.09	1177.75	$[1518.19 - C_{12}H_{24}O_3]^-$
	1132.07	1131.71	$[1348.15 - C_{10}H_{18}O_2]^-$
	1114.07	1113.70	$[1302.12 - C_{10}H_{18}O_2]^-$
	961.98	961.58	$[1302.12 - C_{10}H_{20}O_3]^-$
	944.04	943.57	$[1330.14 - C_{10}H_{18}O_2\text{-}C_{12}H_{22}O_2]^-$

Table S12. Observed peaks in the negative MALDI-QIT-TOF spectrum on MS^4 of m/z 1348.15

	Observed peaks (m/z)	Calculated peaks (m/z)	Interpretation of peaks
MS^4 of m/z 1348.15 parent ion	1178.07	1177.75	$[1348.15 - C_{10}H_{18}O_2]^-$
	1160.06	1159.74	$[1348.15 - C_{10}H_{20}O_3]^-$
	1132.06	1131.71	$[1348.15 - C_{12}H_{24}O_3]^-$
	962.00	961.58	$[1160.06 - C_{12}H_{22}O_2]^-$
	944.02	943.57	$[1160.06 - C_{12}H_{24}O_3]^-$

Supplementary figures

Fig. S1. Location of Midtre Lovenbreen close to Ny-Alesund, in Svalbard, Norway and exact sampling sites marked in red color circles.

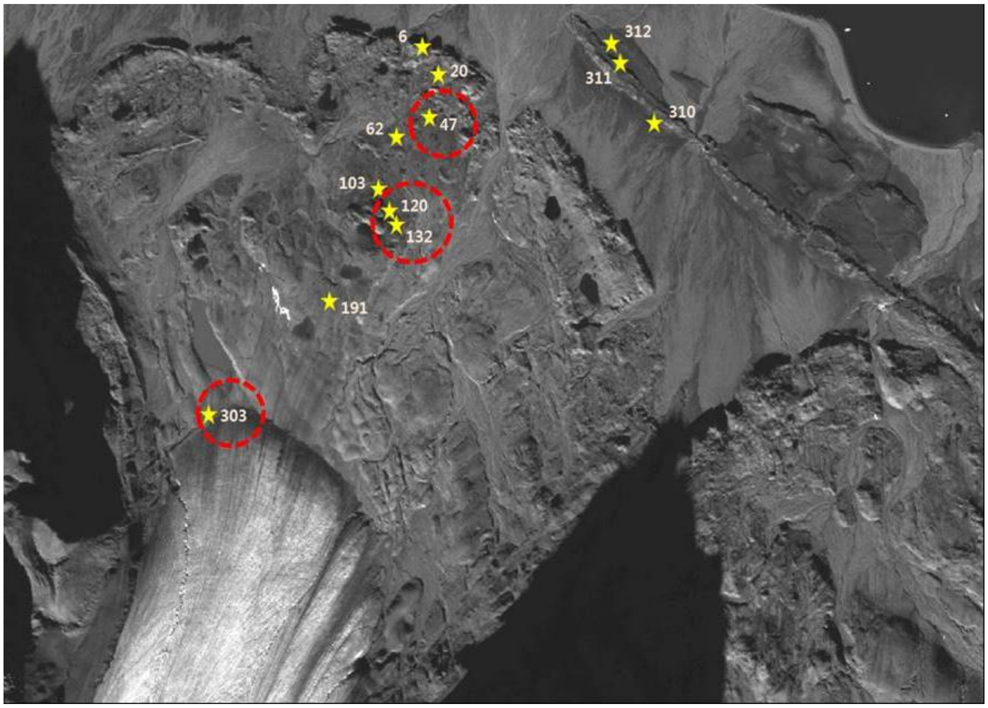

Fig. S2. Fragmentation mechanisms of charge-driven process (A) and charge-remote (B).

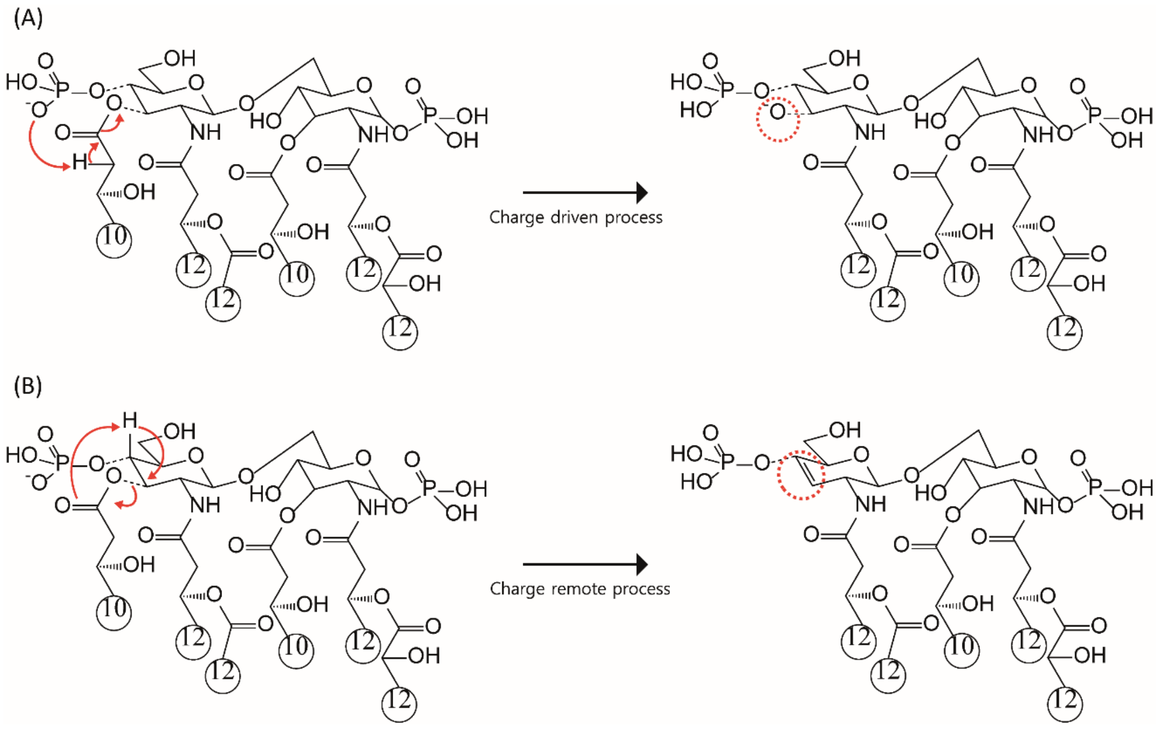

Fig. S3. Standard calibration curve of endotoxin, which shows a correlation between the optical density (OD) measured at 405 nm and endotoxin unit (EU) per mL., Activity of LPS in Pseudomonas sp. strain PAMC 28618 (12 CFU) was approximatively calculated as 0.38 EU/mL (red spot).

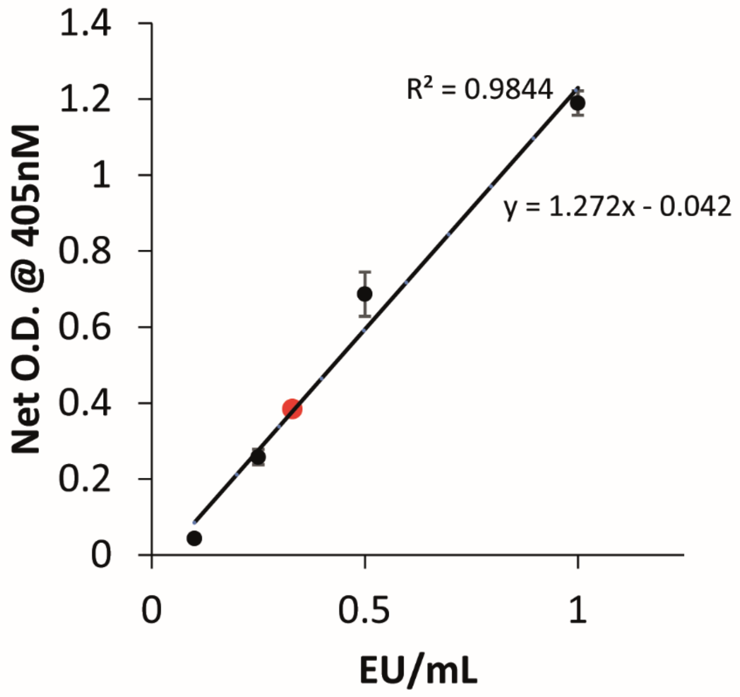

Fig. S4. Negative-ion MALDI-TOF MS spectrum of De-*O*-acylated lipid A from *Pseudomonas* sp. strain PAMC 28618 (A) and *Pseudomonas cichorii* (B)

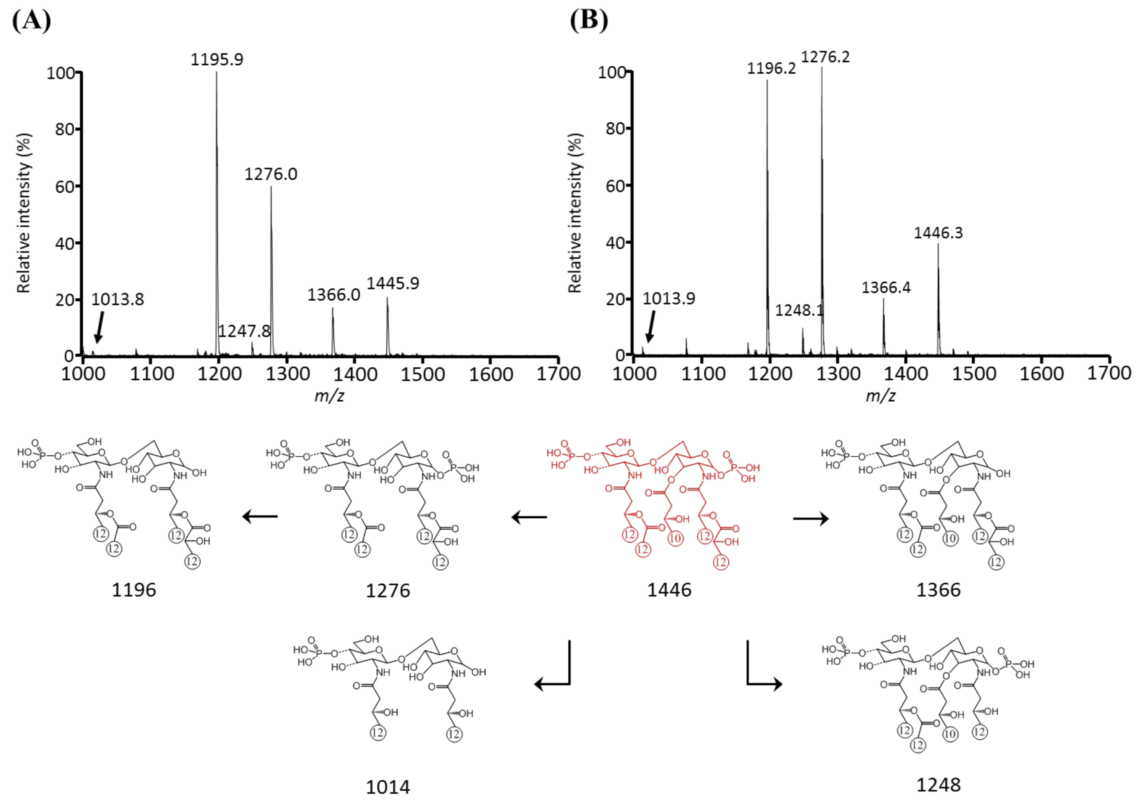

Fig. S5. Another pathway of MS^5 analysis of lipid A from *Pseudomonas* sp. strain PAMC 28618 using MALDI-QIT-TOF MS. Negative-ion MALDI-QIT-TOF MS^3 spectrum of *m/z* 1428.19 (A) and fragment structure (B).

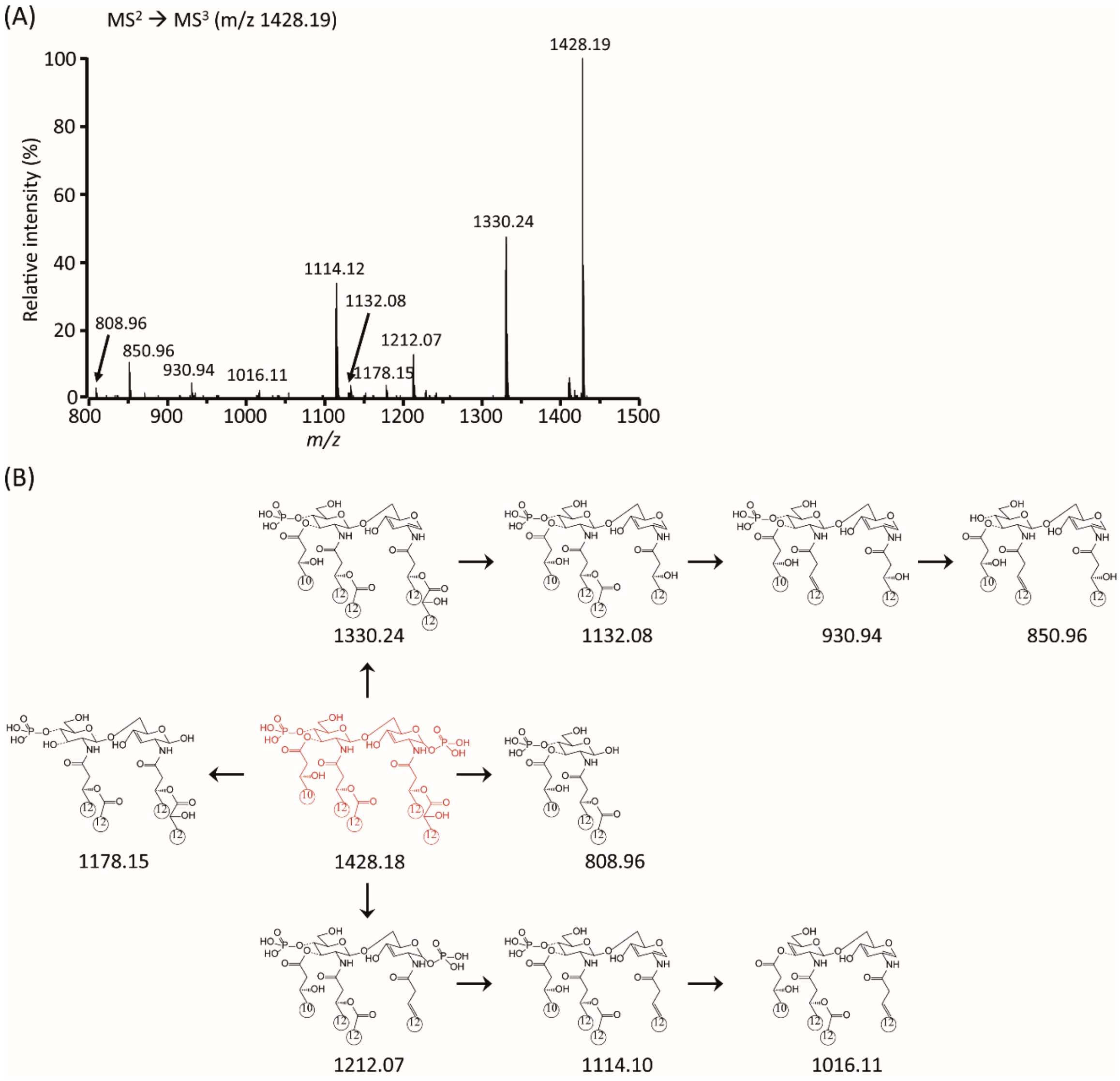

Fig. S6. Negative-ion MALDI-QIT-TOF MS^4 profile of *m/z* 1330.24 of lipid A from *Pseudomonas* sp. strain PAMC 28618 (A) and fragment structure (B).

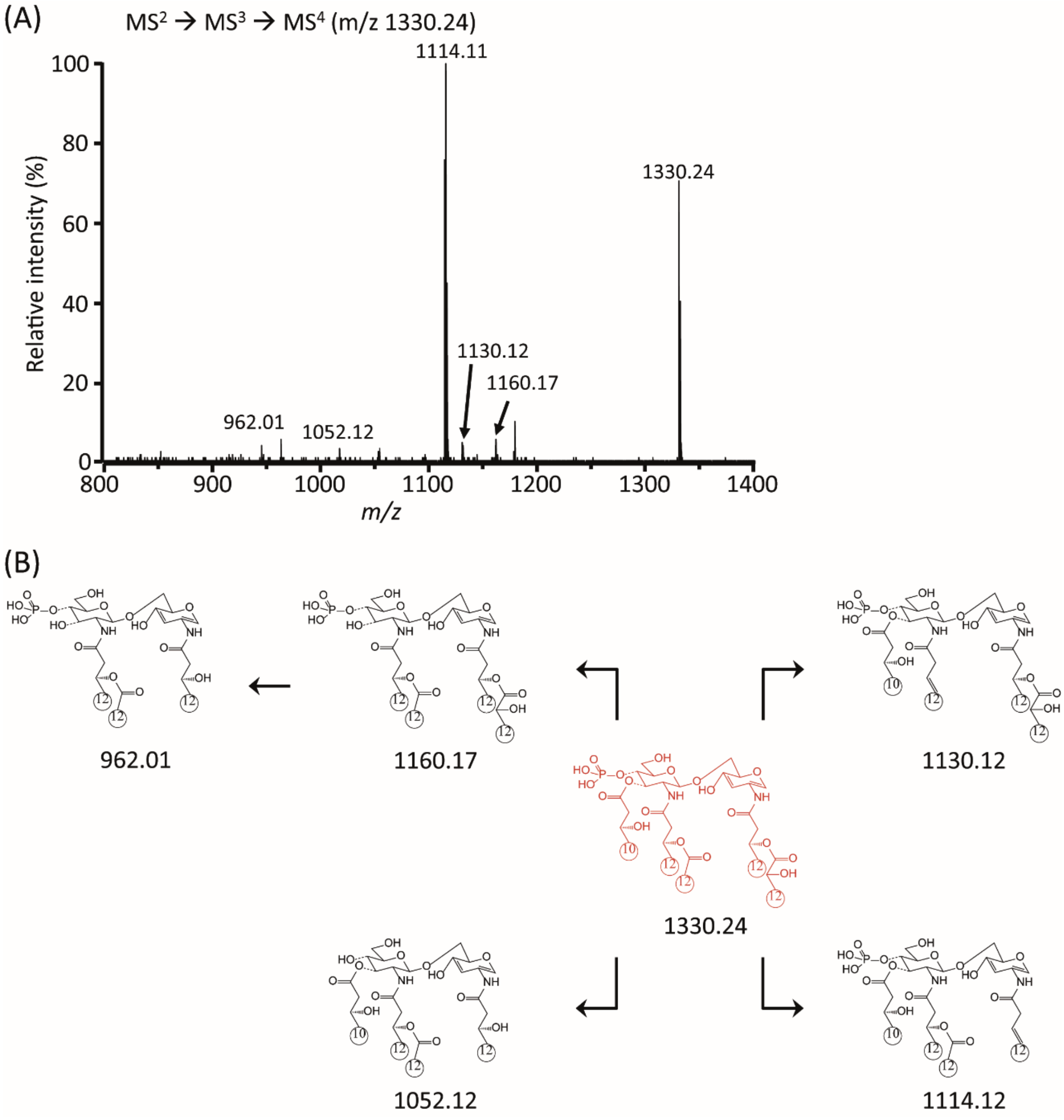

Fig. S7. Negative-ion MALDI-QIT-TOF MS[5] profile of *m/z* 1114.13 of lipid A from *Pseudomonas* sp. strain PAMC 28618 (A) and fragment structure (B).

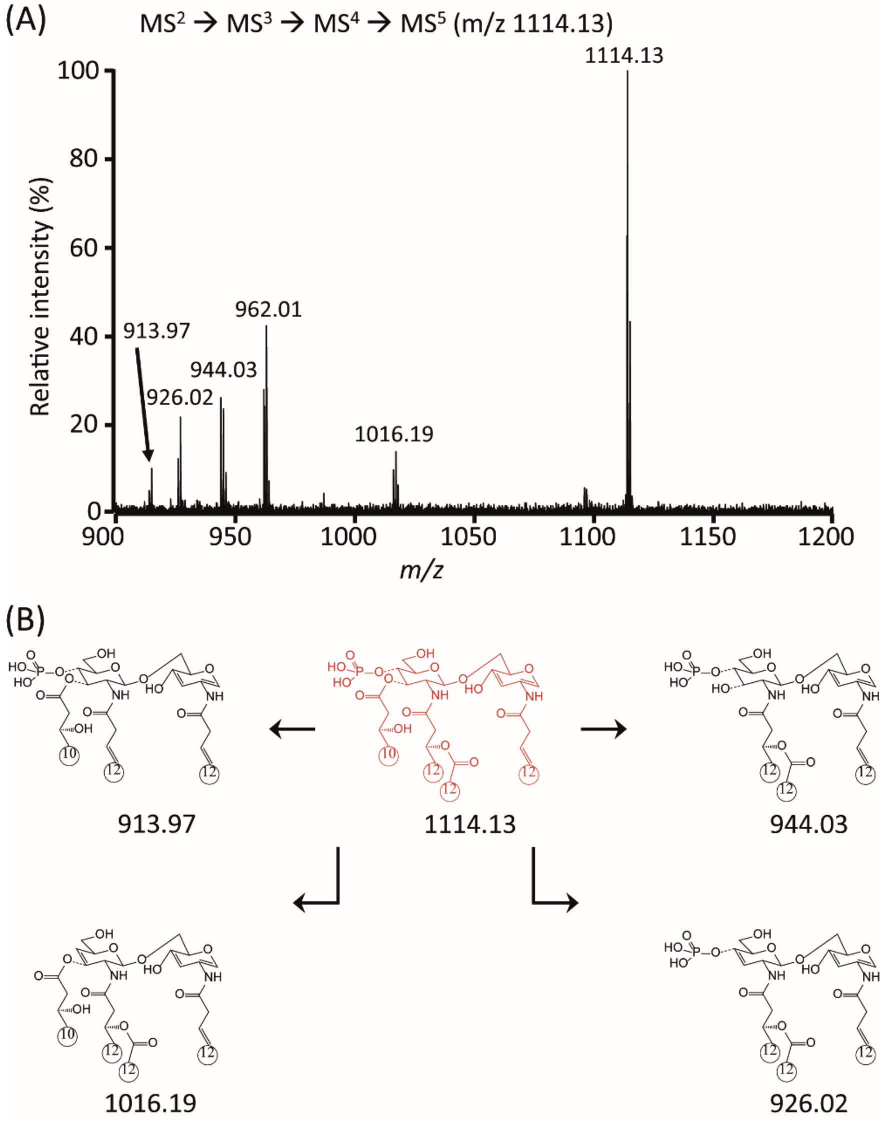

Fig. S8. Negative-ion MALDI-TOF MS (A) and MALDI-QIT-TOF MS (B) mass spectra of lipid A from *Pseudomonas cichorii*.

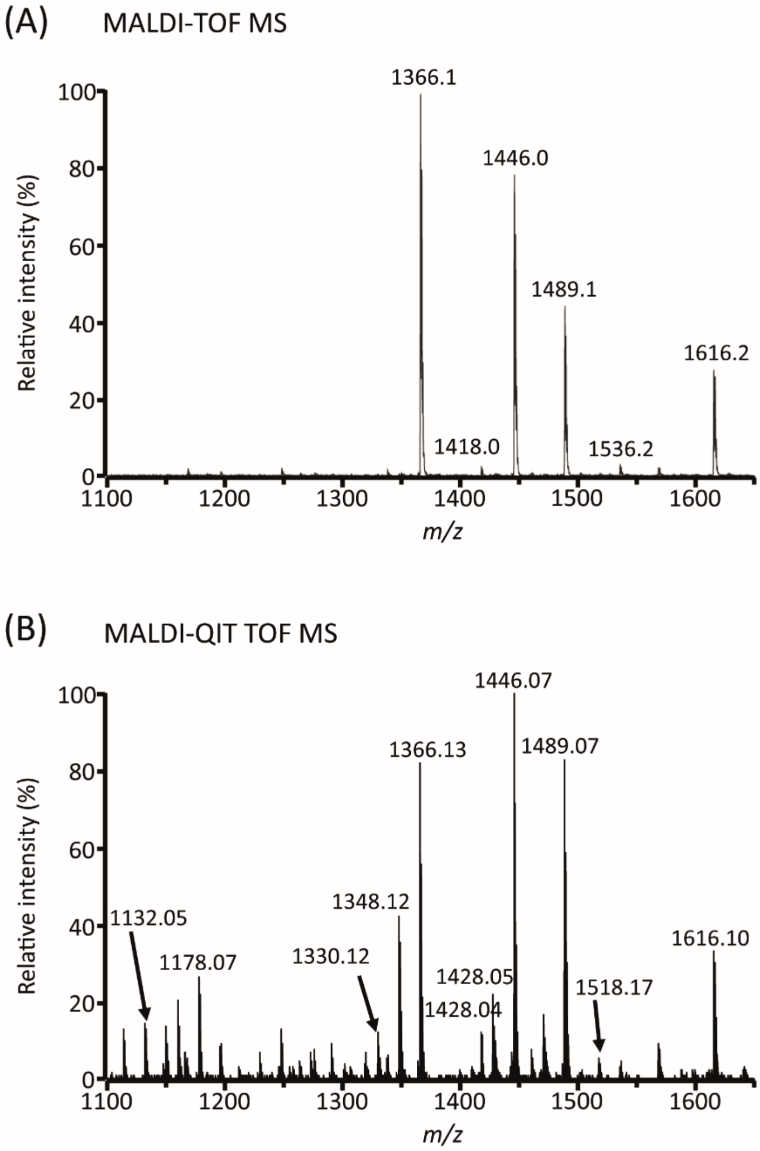

Fig. S9. Negative-ion MALDI-QIT-TOF MS^2 spectrum of *m/z* 1616.10 of lipid A from *Pseudomonas cichorii*; the structures were drawn as neutral molecules.

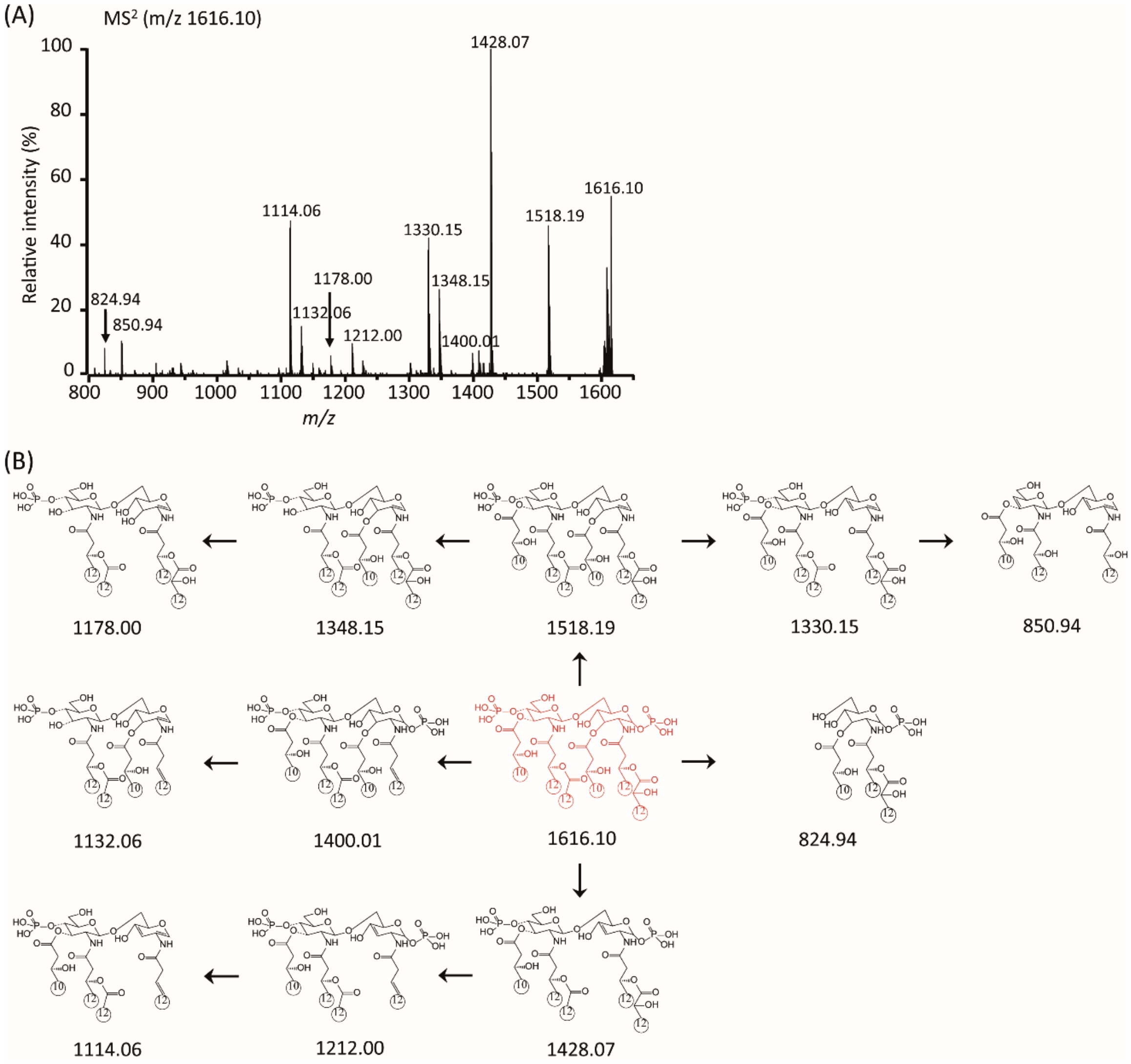

Fig. S10. Negative-ion MALDI-QIT-TOF MS^3 spectrum of *m/z* 1518.19 of lipid A from *Pseudomonas cichorii* (A) and fragment structure (B).

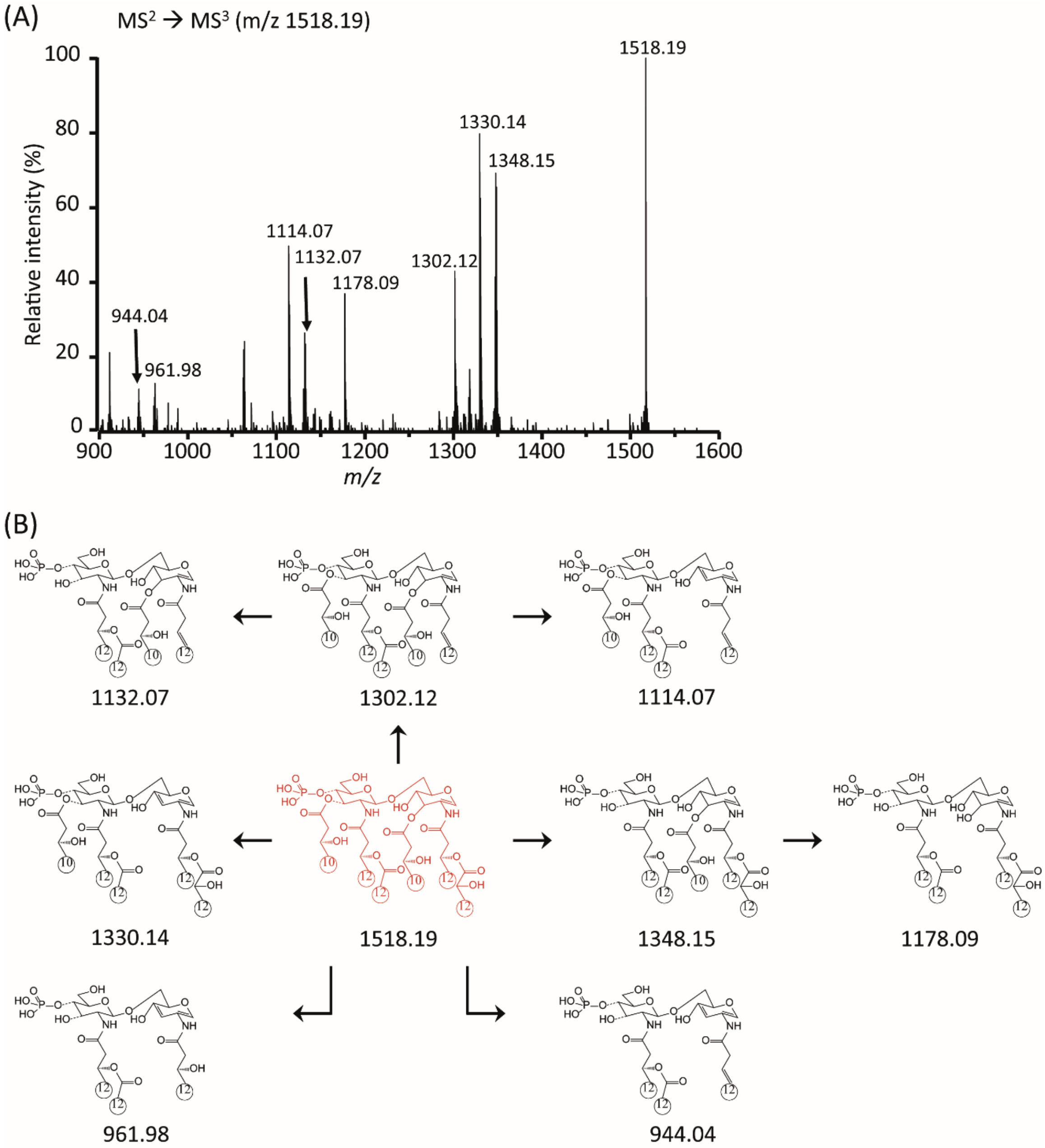

Fig. S11. Negative-ion MALDI-QIT-TOF MS^4 spectrum of *m/z* 1348.15 of lipid A from *Pseudomonas cichorii* (A) and fragment structure (B).

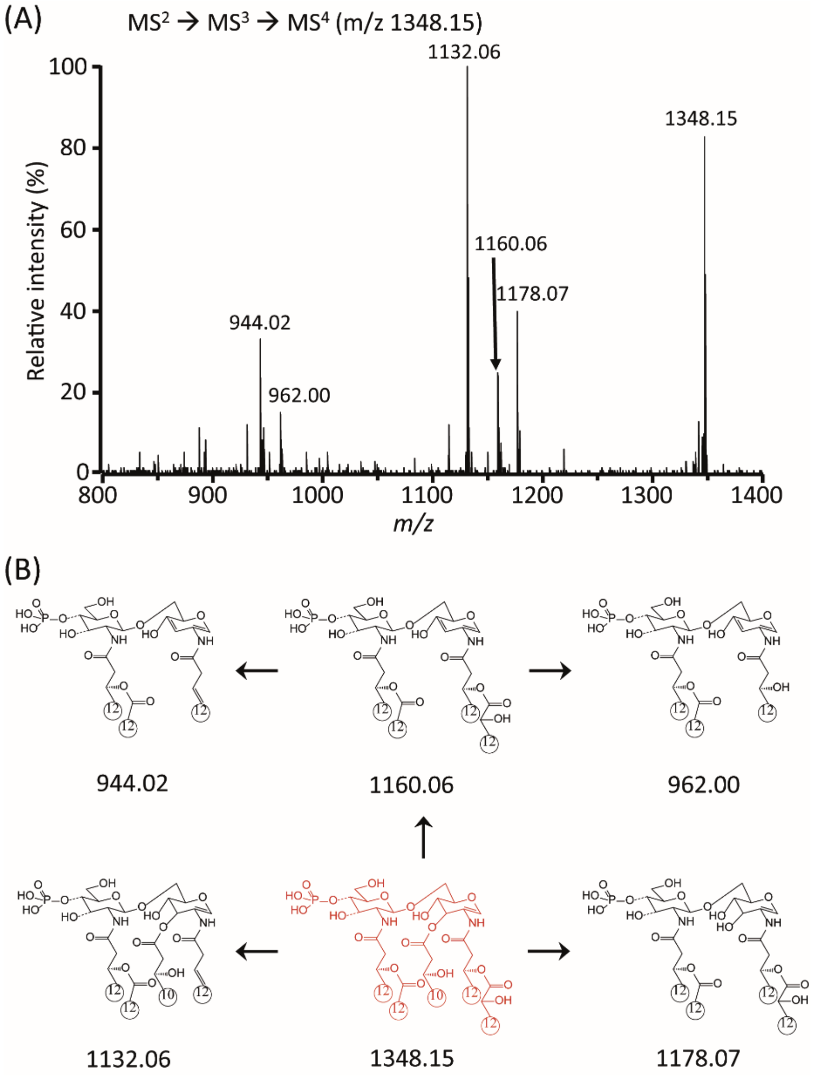

Supplement: Supplementary file 1 — Supplementary Information [file 41598_2017_2145_MOESM1_ESM.pdf]
